# Long-lasting beneficial effects of central serotonin receptor 7 stimulation in female mice modeling Rett syndrome

**DOI:** 10.3389/fnbeh.2015.00086

**Published:** 2015-04-14

**Authors:** Bianca De Filippis, Valentina Chiodi, Walter Adriani, Enza Lacivita, Cinzia Mallozzi, Marcello Leopoldo, Maria Rosaria Domenici, Andrea Fuso, Giovanni Laviola

**Affiliations:** ^1^Department of Cell Biology and Neuroscience, Istituto Superiore di SanitàRome, Italy; ^2^Department of Therapeutic Research and Medicines Evaluation, Istituto Superiore di SanitàRome, Italy; ^3^Department of Pharmacy, University of Bari “A. Moro”Bari, Italy; ^4^Department of Psychology, Section of Neuroscience, Sapienza University of RomeRome, Italy; ^5^European Center for Brain Research (CERC)/IRCCS Santa Lucia FoundationRome, Italy

**Keywords:** serotonin, neurodevelopmental disorders, intellectual disability, transgenic mice, synaptic plasticity, Rho GTPases, cognition

## Abstract

Rett syndrome (RTT) is a rare neurodevelopmental disorder, characterized by severe behavioral and physiological symptoms. Mutations in the methyl CpG binding protein 2 gene (*MECP2*) cause more than 95% of classic cases, and currently there is no cure for this devastating disorder. Recently we have demonstrated that specific behavioral and brain molecular alterations can be rescued in MeCP2-308 male mice, a RTT mouse model, by pharmacological stimulation of the brain serotonin receptor 7 (5-HT7R). This member of the serotonin receptor family—crucially involved in the regulation of brain structural plasticity and cognitive processes—can be stimulated by systemic repeated treatment with LP-211, a brain-penetrant selective 5-HT7R agonist. The present study extends previous findings by demonstrating that the LP-211 treatment (0.25 mg/kg, once per day for 7 days) rescues RTT-related phenotypic alterations, motor coordination (*Dowel test)*, spatial reference memory (*Barnes maze* *test*) and synaptic plasticity (*hippocampal long-term-potentiation*) in MeCP2-308 heterozygous female mice, the genetic and hormonal milieu that resembles that of RTT patients. LP-211 also restores the activation of the ribosomal protein (rp) S6, the downstream target of mTOR and S6 kinase, in the hippocampus of RTT female mice. Notably, the beneficial effects on neurobehavioral and molecular parameters of a seven-day long treatment with LP-211 were evident up to 2 months after the last injection, thus suggesting long-lasting effects on RTT-related impairments. Taken together with our previous study, these results provide compelling preclinical evidence of the potential therapeutic value for RTT of a pharmacological approach targeting the brain 5-HT7R.

## Introduction

The serotonin receptor 7 (5-HT7R), coded by Htr7 gene, is among the most recently discovered serotonin receptors (Barnes and Sharp, 1999). This seven-transmembrane and G protein-coupled receptor is characterized by a widespread expression in the central nervous system and in the periphery (Romano et al., 2014). The highest 5-HT7 receptor density in the brain is found in the thalamus and hypothalamus, as well as in the hippocampus (Guseva et al., 2014). Thanks to the availability of pharmacological and genetic tools targeting the 5-HT7R in preclinical models (Leopoldo et al., 2011; Matthys et al., 2011), a link with neuro-physiological phenomena like regulation of the circadian rhythm, sleep, mood and thermoregulation has been clearly established (Matthys et al., 2011; Adriani et al., 2012; Monti and Jantos, 2014). The relevance of the 5-HT7R in various psychiatric and neurological disorders, such as anxiety, schizophrenia, pain and epilepsy has been also addressed (Hedlund, 2009; Di Pilato et al., 2014). An increasing number of studies demonstrates a role for the 5-HT7R on cognitive processes (particularly on hippocampal-dependent learning and memory) and in the regulation of structural plasticity in adolescent and mature brain circuits (Gasbarri and Pompili, 2014; Meneses, 2014; Volpicelli et al., 2014; Canese et al., 2015). Consistent with these observations, the 5-HT7R activation stimulates signaling cascades known to play a prominent role in synaptic plasticity and cognition, with the more prominent downstream effectors being represented by the extracellular-signal regulated kinases (ERKs), the cyclic AMP protein kinase (PKA) and the Cyclin-dependent kinase 5 (Cdk5; Guseva et al., 2014; Volpicelli et al., 2014).

In cultured hippocampal neurons, receptor-mediated activation of the Gα12 signaling pathway also results in the selective activation of small Rho GTPases (Kvachnina et al., 2005; Kobe et al., 2012), a family of proteins crucially involved in neuronal plasticity and cognition and key regulators of actin cytoskeleton dynamics (De Filippis et al., 2014b). The 5-HT7R-mediated stimulation of this signaling pathway leads to pronounced changes in neuronal morphology and plasticity (Kobe et al., 2012), thus providing further support to a crucial involvement of 5-HT7R in the regulation of neurobiological mechanisms underlying cognitive functions (Volpicelli et al., 2014). Recently, we have extended these findings by providing the first *in vivo* evidence that a pharmacological stimulation of the 5-HT7R (i.e., by systemic administration of LP-211, a novel selective and brain penetrant 5-HT7R agonist: see (Hedlund et al., 2010; Romano et al., 2014), activates Rho GTPases in mouse brain (De Filippis et al., 2014a). Given the involvement of brain Rho GTPases in a number of neurological disorders and the paucity of drugs targeting this family of proteins *in vivo* (De Filippis et al., 2014b), these data pointed to LP-211 as an innovative pharmacological tool to be exploited in preclinical research. In this line, activation of 5-HT7Rs has been reported to reverse electrophysiological abnormalities in hippocampal slices collected from a mouse model of Fragile X syndrome (Costa et al., 2012).

Based on these findings, we have recently investigated the potential therapeutic value of a pharmacological stimulation of the central 5-HT7R for Rett syndrome (RTT), a rare and severe neurodevelopmental disorder (Percy and Lane, 2005) and one of the leading causes of mental disability in girls (Rett, 1966; Hagberg, 2002). RTT patients present stereotypical hand movements, limited language, severe autistic−like features and intellectual disability, as well as potentially life−threatening seizures and respiratory dysfunction. Mutations in the methyl-CpG-binding protein 2 (MeCP2) gene, located on Xq28, have been identified as the main genetic cause of RTT (Amir et al., 1999) and account for more than 95% of classic RTT (Chahrour and Zoghbi, 2007). At present there is no cure for RTT and available treatments are symptomatic.

Consistent with previous evidence pointing to Rho GTPases as therapeutic targets for RTT (De Filippis et al., 2012) and with previous studies suggesting that serotonergic neurotransmission is deeply affected both in RTT patients and animal models (Isoda et al., 2010; Santos et al., 2010; Moroto et al., 2013), we demonstrated that a seven-day-long treatment with LP-211 reverses anxiety-related profiles in a Light/Dark test, motor abilities in a Dowel test, the exploratory behavior in the Marble Burying test, as well as short-term working memory in the Y maze task in MeCP2-308 hemizygous (hz) male mice (Ricceri et al., 2013). Moreover, this treatment was found to restore the activation of Rho GTPases effector molecules, PAK, cofilin and the ribosomal protein (rp) S6, a downstream target of mTOR and S6 kinase (Ricciardi et al., 2011), in RTT mouse hippocampus (De Filippis et al., 2014a).

In the present study, we aimed at assessing whether the beneficial effects of the LP-211 treatment in RTT mice extend beyond the behavioral domains we had previously investigated (De Filippis et al., 2014a). To increase the translational value of the study, we focused on MeCP2-308 heterozygous (Het) female mice, given that their genetic and hormonal milieu more closely resembles that of RTT patients (Katz et al., 2012). Increasing studies in fact demonstrate that heterozygosity does not preclude the use of female mice in RTT preclinical studies (Woods et al., 2012; Garg et al., 2013). Therefore, in symptomatic MeCP2-308 Het female mice, we have evaluated whether treatment with LP-211 affects spatial reference memory deficits in the Barnes Maze test and impairments of hippocampal long-term potentiation (LTP), a form of synaptic plasticity thought to underline long-term memory formation. Given the demonstrated role played by 5-HT7R in the regulation of cognitive performance and synaptic plasticity (Gasbarri and Pompili, 2014; Meneses, 2014; Volpicelli et al., 2014), we argued that 5-HT7R stimulation by LP-211 in RTT mouse brain might restore these additional RTT-related deficits. Moreover, in order to confirm data obtained in LP-211-treated MeCP2-308 male mice (De Filippis et al., 2014a) and assess whether this treatment is equally effective under conditions of heterozygosis, the effects of the treatment on motor coordination deficits in RTT female mice were also addressed with the Dowel test.

Another major aim of the present study was to discover whether a seven-day-long treatment with LP-211 may produce long-lasting changes in the neurobehavioral phenotype or at the brain molecular level in RTT mice. This hypothesis stems from previous studies demonstrating that transient modulation of Rho GTPases in RTT mouse brain produces long-lasting beneficial effects (De Filippis et al., 2012) and that 5-HT7 stimulation during periods of increased plasticity, such as adolescent age, may result in neuro-plastic changes leading to a persistent modification on forebrain circuits (Adriani et al., 2006; Altabella et al., 2014; Canese et al., 2015). To evaluate this hypothesis, we applied the seven-day-long treatment schedule known to induce Rho GTPases activation in mouse brain (De Filippis et al., 2014a) and tested the effects on general health parameters as well as behavioral, molecular and synaptic plasticity endpoints up to 2 months after the last injection).

## Materials and Methods

### Subjects

The experimental subjects were 1-year old MeCP2-308 heterozygous female mice [B6.129S-MeCP2tm1Heto/J, stock number: 005439; backcrossed to C57BL/6J mice for at least 12 generations from the Jackson Laboratories (USA)] and wild-type (wt) littermates. The MeCP2-308 model bears a truncating mutation, leading to the expression of a protein truncated at amino acid 308 (Shahbazian et al., 2002). In agreement with clinical data from RTT patients carrying C-terminal deletions of the MeCP2 gene (Díaz de León-Guerrero et al., 2011), this model presents a delayed onset of symptoms and a prolonged life-span in comparison with knockout mice (Ricceri et al., 2008).

Mice were housed in groups of 2–3 in polycarbonate transparent cages (33 × 13 × 14 cm) with sawdust bedding and kept on a 12-h light-dark schedule (lights off at 8:00). Temperature was maintained at 21 ± 1°C and relative humidity at 60 ± 10%. Animals were provided *ad libitum* with tap water and a complete pellet diet (Altromin, Germany). All procedures were carried out in accordance with the European Communities Council Directive (86/609/EEC) as well as Italian law, and formally approved by Italian Ministry of Health.

### Drug and Treatment

LP-211 was prepared following the same synthetic procedure described in Leopoldo et al. (2008). The compound was dissolved in a vehicle solution of 1% dimethyl sulfoxide (DMSO) in saline (0.9% NaCl). MeCP2-mutated mice and wt littermate controls were randomly assigned to be daily intra-peritoneally (ip) injected (between 9.00 and 11.00 am) for 7 consecutive days with either LP-211 (0.25 mg/kg) or vehicle (1% of DMSO in saline). The number of mice for each condition was as follows: wt, Veh = 9; wt, LP-211 = 11; Het, Veh = 12; Het, LP-211 = 10.

To test whether LP-211 can counteract RTT related abnormalities when they are fully manifested, MeCP2-308 heterozygous female mice were treated at about 1 year of age, when abnormalities have been reported to start appearing (Shahbazian et al., 2002).

### Behavioral Testing

**Figure 1:**
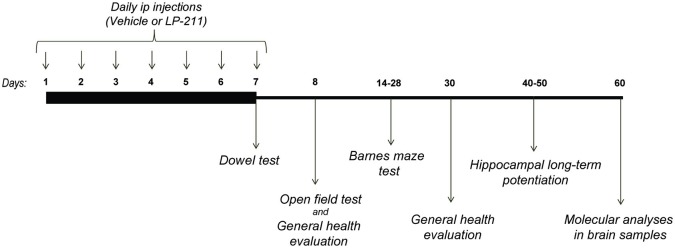
**Experimental Design**.

Mice were experimentally naïve at the start of the test battery. All behavioral testing took place during the dark phase of the L/D cycle, between 9.00 am and 6.00 pm, and was carried out by experimenters blind to mouse genotypes and treatments. The estrous cycle was not controlled in this study (Prendergast et al., 2014). A minimum of 24 h was left between each test, as follows: the *Dowel test* was performed 2 h after the 7th ip injection; the *open field test* was carried out 24 h after the last ip injection (on the 8th day of the schedule); the *general health scoring* was carried out after the *open field test* on the 8th day of the schedule and 23 days after the last ip injection (on the 30th day of the schedule); the training on the *Barnes Maze test* started on the 14th day of the schedule and the probe tests were conducted on the 21st and 28th days of the schedule (see Figure 1 for experimental design and treatment schedule).

#### General Health Scoring

The general health of the experimental mice was qualitatively evaluated by a trained observer as previously described (Guy et al., 2007; De Filippis et al., 2014a), with little modification. Briefly, mice received a score (ranging from 0: normal appearance, to 2: highly compromised) for each of the following symptoms: gait, mobility, breathing, kyphosis, fur, hindlimb clasping, tremors, general condition. The individual scores for each of these categories were subsequently averaged to obtain a semi-quantitative measure of individual symptom status, called throughout the text “*the general health score*”.

Body weight and body (rectal) temperature were recorded after each general health scoring.

#### Dowel Test

To evaluate the effects of the LP-211 treatment on motor coordination capacities, the *Dowel test* was performed as previously described (De Filippis et al., 2014a). The hardwood round dowel used was 9.0 mm in diameter and 35 cm long. The dowel was mounted horizontally 50 cm above a 5 cm depth bedding of sawdust. At the beginning of the each testing session, each mouse was placed in the middle of the dowel so that the length of its body was parallel to it. Latency to fall from the dowel into a cage of bedding was recorded (30-s criterion). Each mouse repeated the test three times, with an intertrial interval of at least 15 min. If mice were able to walk across the dowel and off of the dowel, they received the maximum score of 30 s.

#### Open Field Test

The *open field* apparatus was a gray plastic box (40 × 40 cm) surrounded by high walls (35 cm). Each mouse was individually placed in the *open field* and allowed to freely explore the environment for a 60 min session (De Filippis et al., 2013). The floor of the apparatus was cleaned with 20% ethanol after each animal was tested and the test was carried out under dim lights. Three intervals of 5 min (0–5; 30–35; 55–60) were subsequently scored by a trained observer blind to the genotype and treatment of mice, using a computer and a specific software (THE OBSERVER v2.0 for DOS, Noldus Information Technology, Wageningen, Netherlands). The floor of the apparatus was subdivided into 16 sections (10 × 10 cm) by lines placed on the video screen at the time of videotape analysis. The frequency and durations of the following items were scored: *Crossing* (number of line crossings with both forepaws), *Rearing* (body in vertical position), *Wall rearing* (body in vertical position with forepaws placed on the walls of the cage), *Grooming* (mouth or paws on body) and *Inactivity* (complete absence of movements including small movements of head, ears or vibrissae).

#### Barnes Maze Test

To assess whether the treatment with LP-211 has beneficial effects on spatial reference memory deficits in RTT mice, the *Barnes Maze test* was carried out as previously described (Barnes, 1979).

In this test, mice were trained to locate a black rectangular escape box (7 × 37 × 9 cm) hidden underneath one of 12 holes (4 cm in diameter) evenly spaced around the perimeter of an elevated (36 cm above the floor) gray platform (95 cm in diameter), illuminated by overhead fluorescent white room lighting (85 lux). The hole above the escape box was designated as the target, analogous to the hidden platform in the Morris water maze task. The location of the target was consistent for a given mouse but randomized across mice. To prevent orientation to the target before a trial began, mice were initially placed in the center of the platform under a black cylinder (12 cm in diameter). The cylinder was removed after 10 s and the trial begun. The maze was cleaned with a 20% ethanol solution between each trial.

Behavioral testing consisted of an adaptation period, an acquisition phase and 2 probe trials. During the adaptation phase (14th day of the schedule), mice were allowed to explore for two consecutive daily trials the platform. During each trial, if 1 min had elapsed without the mice entering the target hole, they were placed to the side of the target hole and gently helped to enter the escape box. Once inside the escape box, the mouse was left there for 2 min. No parameters were recorded during this phase.

During the acquisition phase (from the 15th to the 20th day of the schedule), mice were given two trials per day during which latency to enter the target hole and total path length were recorded. When the trial ended (i.e., when the mouse entered the escape box or after 3 min had elapsed), the mouse was left inside the escape box for 1 min. An intertrial interval of at least 10 min was used. During the acquisition phase, latency, errors and path length to enter the target hole were measured. For statistical analyses parameters were averaged in blocks of trials per day (mean ± S.E.M).

The first probe trial was conducted 24 h after the last training trial to assess short-term reference memory retention (21th day of the schedule). To assess long-term retention a second probe trial was applied 7 days after the first probe (28th day of the schedule). No training trials were conducted between the two probe tests. During the probe tests, the target hole was closed to confirm that mice used only extra-maze cues to reach the escape box. A 90-s long session was used, during which primary latency (to first nose poke in the virtual target hole) and primary errors (i.e., nose pokes before arriving to the target) were measured.

### Neurobiological Analyses

Starting on the 40th day of the schedule, mice underwent electrophysiology experiments (*N* = 4 mice per experimental group). The brains of the remaining subjects were dissected and rapidly frozen for biochemical analyses at sacrifice, 2 months after the last ip injection.

#### Hippocampal Slice Electrophysiology

To evaluate the induction of LTP in wt and MeCP2-308 female mice, hippocampal slices were prepared as previously described (Domenici et al., 2006). Field excitatory post-synaptic potentials (fEPSPs) were recorded in stratum radiatum of CA1 area after stimulation of the Schaffer collaterals. Traces were acquired, amplified and analyzed with DAM-80 AC differential amplifier (WPI Instruments) and with the WinLTP software (Anderson and Collingridge, 2007). Stimuli (100 μs duration) were set to an intensity that evokes a fEPSP with a slope of 60% of the maximum fEPSP slope and delivered every 20 s (three consecutive responses were averaged). LTP was induced by a theta-burst stimulation (TBS) consisting in 2 trains of 5 sets of bursts (four stimuli, 100 Hz) with an interburst interval of 200 ms and a 20 s interval between each train. Synaptic transmission was recorded for 60 min and 10 min of stable baseline recordings preceded LTP induction. Changes in fEPSP slope were expressed as percentage changes with respect to the average slope of the fEPSP measured during the 10 min that preceded the TBS.

#### Western Blot Analyses

Proteins were analyzed by western blotting as previously described (De Filippis et al., 2014a). Briefly, hippocampal tissues were isolated and homogenized in lysis buffer immediately after sacrifice, proteins were separated by SDS-PAGE and blotted to nitrocellulose membrane. The following primary antibodies were used: rabbit polyconal anti-rpS6 (1:1000, cod. 2217, Cell Signaling), rabbit polyclonal anti-phospho-rpS6 (1:200, cod. 2211 (Ser 235/236) and cod. 2215 (Ser 240/244), Cell Signaling), mouse monoclonal anti β-actin (1:1000, cod. sc-81178, Santa Cruz). Optical densities (O.D.) of the protein signals from at least three different experiments were calculated for each sample and normalized with the corresponding β-actin signal; the O.D. ratios were then compared and expressed as the average fold increase, with 1 (wt control) as baseline.

### Statistical Analysis

Data were analyzed with either parametric or non-parametric analysis of variance, depending on distribution of the response variable considered. The Shapiro-Wilk test was applied to verify the normality of data distribution. ANOVA models included genotype (wt vs. Het) and treatment (vehicle vs. LP-211) as between-subject factors, and repeated measurements as within-subject factor. *Post hoc* comparisons were run using Tukey’s test, which can be performed also in the absence of significant ANOVA results (Wilcox, 1987). The Levene test was applied to confirm that variance did not differ between groups. To unravel the presence of outliers, the Grubbs’ test was applied.

## Results

### General Health Scoring

**Figure 2:**
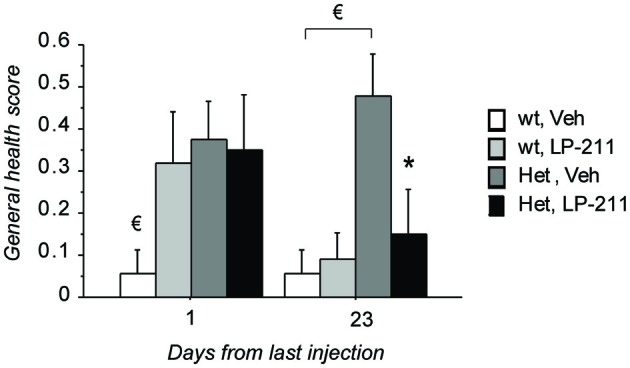
**Treatment with LP-211 exerts delayed beneficial effects on general health parameters in MeCP2-308 female mice**. MeCP2-308 female mice (Het) showed slight but reliable phenotypic alterations compared to wt mice in control conditions (Veh). The seven-day long LP-211 treatment improved the general health status selectively in Het mice (Het, LP-211) after 3 weeks from the last injection. Mice for each condition were as follows: wt, Veh = 9; wt, LP-211 = 11; Het, Veh = 12; Het, LP-211 = 10. Data are mean ± SEM. € : wt, Veh vs. Het, Veh, *p* < 0.05 and * : Het, Veh vs. Het, LP-211, *p* < 0.05.

Kruskall Wallis analyses confirmed that significant differences among the experimental groups are present on both time points under investigation (first time interval: *H* = 8.89; *p* = 0.031; second time interval: *H* = 9.31; *p* = 0.025; Figure 2). *Post hoc* comparisons, carried out with the Mann Whitney U test, revealed that—at 24 h after the last ip injection—slight but reliable phenotypic alterations can be detected in MeCP2-308 female mice compared to wt controls (wt, Veh vs. Het, Veh: *U* = 23.50; *p* = 0.030; Figure 2). In particular, the phenotypic parameters which appeared most compromised in MeCP2-308 female mice were the gait, the kyphosis and breathing. Contrary to MeCP2-null mice (Guy et al., 2007), no hindlimb clasping or abnormal mobility were observed in RTT mice vs. wt controls. At this point of the treatment schedule (Figure 1), no recovery in the general health status was observed for RTT mice treated with LP-211 compared to vehicle, with both being significantly worse than wt controls (Figure 2). Notably, however, recovery was observed 3 weeks later: MeCP2-308 female mice that had received the vehicle treatment were slightly worsened (wt, Veh vs. Het, Veh: *U* = 18.00; *p* = 0.011), whereas RTT mice that were treated with LP-211 exhibited an improved general health status (Het, Veh vs. Het, LP-211: *U* = 29.00; *p* = 0.041; Figure 2). A delayed effect of the 7-day long LP-211 treatment on general health status of RTT mice was thus demonstrated.

No significant genotype nor treatment effects were found as for body weight and body temperature (data not shown).

### Dowel Test

**Figure 3:**
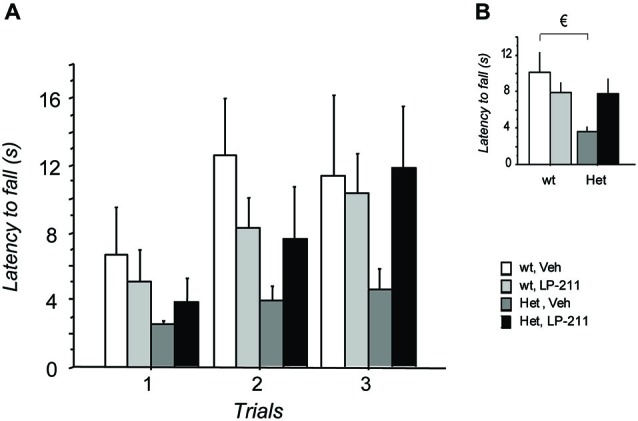
**Treatment with LP-211 improves motor coordination capacities of MeCP2-308 female mice in the Dowel test. (A)** In the Dowel test, MeCP2-308 female mice (Het, Veh) displayed significantly shorter latencies to fall compared to wt controls (wt, Veh), confirming impaired motor coordination capacities. LP-211 improved the performance of Het mice in this test. Three trials per mouse were performed, with an intertrial interval of at least 15 min. **(B)** Data from the three trials were pooled to highlight significant differences between the experimental groups. Mice for each condition were as follows: wt, Veh = 9; wt, LP-211 = 11; Het, Veh = 12; Het, LP-211 = 10. Data are mean ± SEM. € : wt, Veh vs. Het, Veh, *p* < 0.05.

Performance in the Dowel test on the 7th day of treatment was investigated to confirm data obtained in LP-211-treated MeCP2-308 hz male mice (De Filippis et al., 2014a). The repeated measures (RM)- ANOVA yielded a significant main effect of genotype (*F*_(1,38)_ = 4.12; *p* = 0.049), with RTT mice falling from the dowel significantly earlier than wt controls (Figure 3), and a genotype by treatment interaction just missing significance (*F*_(1,38)_ = 3.77; *p* = 0.060). *Post hoc* comparisons by Tukey’s test confirmed that the performance of MeCP2-308 heterozygous female mice was worse compared to that of wt controls (*p* < 0.05). A just missing effect of the LP-211 treatment on the motor coordination ability of RTT mice was also found, with the treatment improving their performance (Figure 3). The LP-211 treatment did not affect the performance of wt mice.

### Open Field Test

In order to clarify LP-211 treatment effects on general locomotor activity, MeCP2-308 mice and wt littermates were tested in the *open field* test. The RM- ANOVA did not highlight any difference between groups as for the mean number of *crossings* (wt Veh: 74.78 ± 5.69; wt LP-211: 69.67 ± 4.80; Het Veh: 79.53 ± 5.04; Het LP-211: 75.47 ± 4.63) and *the time mice spent in the central area* of the arena (wt Veh: 29.26 ± 7.18s; wt LP-211: 26.75 ± 5.81; Het Veh: 27.06 ± 3.92; Het LP-211: 22.75 ± 4.20). Mice also spent a similar amount of time being *immobile* or performing self-directed *grooming* (data not shown). Mutant mice however performed a significantly lower number of *rearings* (wt _(veh + LP-211 pooled)_: 34.88 ± 2.08; Het _(veh + LP-211 pooled)_: 25.59 ± 1.36; main effect of genotype: *F*_(1,38)_ = 7.63; *p* = 0.009), thus confirming previous reports (De Filippis et al., 2012). As a whole, the LP-211 treatment did not affect the behavioral profile of either wt or mutant mice in this test.

### Barnes Maze Test

#### Acquisition

As expected, latency to locate (first nose poke) and to enter the target hole decreased across trials during the acquisition phase (main effect of days: *F*_(4,148)_ = 22.78; *p* > 0.001; *F*_(4,148)_ = 24.56; *p* > 0.001, respectively), thus confirming that all the mice learned to locate the target hole across the training sessions.

As a whole, mutant mice did not differ from wt controls during the training trials (no genotype nor genotype by RM interactions were found for any of the analyzed parameters).

#### Probe Tests

**Figure 4:**
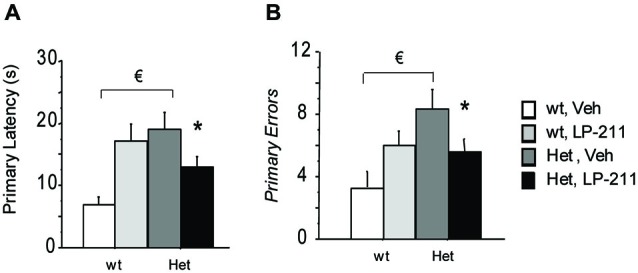
**Stimulation of the 5-HT7 receptor by LP-211 improves spatial memory of MeCP2-308 female mice in the Barnes Maze test**. MeCP2-308 female mice (Het, Veh) displayed longer primary latencies **(A)** and a higher number of primary errors **(B)** to locate the virtual target hole (first nose poke in the virtual target hole) during the Probe tests. LP-211 treatment partially restored the levels of both parameters in Het mice **(A,B)**. Mice for each condition were as follows: wt, Veh = 9; wt, LP-211 = 11; Het, Veh = 12; Het, LP-211 = 10. Data are means ± SEMs. € : wt, Veh vs. Het Veh, *p* < 0.05 and * : Het, Veh vs. Het, LP-211, *p* < 0.05.

On the 21st day (24 h after the last training trial) and the 28th day of the treatment schedule (7 days after the first probe test), the target hole was closed and the probe trials were conducted to assess spatial reference memory retention. Data were analyzed with the RM-ANOVA, with probe tests as within-subject factor. During both probe tests, MeCP2-308 heterozygous female showed a worse performance compared to wt controls, as demonstrated by the longer latency to the first nose poke in the virtual target hole (primary latency) (*p* < 0.05 after *post hoc* comparisons on the genotype by treatment interaction: *F*_(1,37)_ = 7.89; *p* = 0.008; Figure 4A) and the higher number of primary errors RTT mice performed (*p* < 0.05 after *post hoc* comparisons on the genotype by treatment interaction: *F*_(1,37)_ = 4.41; *p* = 0.043; Figure 4B (Moretti et al., 2006). Importantly, LP-211 was able to reverse this impairment and restored wt-like levels of both parameters (*p* < 0.05; Figure 4).

An increase in both parameters, suggestive of a worse performance in this cognitive test, was also evident in wt mice treated with LP-211 compared to veh-treated controls (*p* < 0.05). Interestingly, during both probe tests all the experimental groups showed a comparable profile. Consistently, the RM-ANOVA did not yield significant effects of repeated measurements (Probe 1 and Probe 2), or genotype by RM or treatment by RM interactions.

### Electrophysiology Experiments

**Figure 5:**
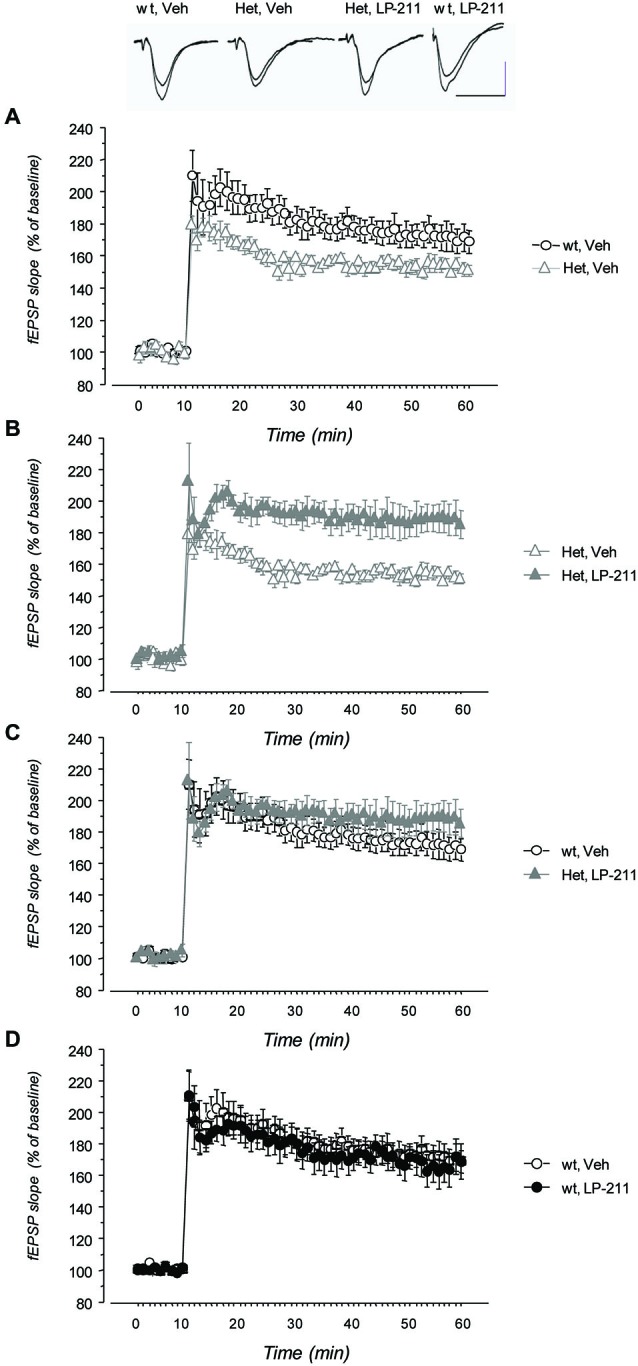
**Treatment with LP-211 improves long-term potentiation (LTP) in MeCP2-308 female mice**. Field EPSPs (fEPSPs) were recorded in the CA1 area of hippocampal slices; LTP was induced by theta burst stimulation (TBS) of Schaffer collaterals. **(A)** MeCP2-308 female mice (Het, Veh) showed a reduced LTP compared to wt littermate controls (wt, Veh). **(B)** The LP-211 treatment significantly increased the response to TBS in MeCP2-308 mice (Het, LP-211) with respect to Het controls. **(C)** Comparison of the time courses of fEPSP slope after TBS did not show significant differences between LP-211-treated Het and wt mice, thus confirming a rescue of LTP in Het mice treated with LP-211. **(D)** LP-211 treatment did not affect the response to TBS in wt mice (wt, LP-211). Data are mean ± S.E.M. The number of slices for each condition was as follows: wt, Veh = 6; wt, LP-211 = 7; Het, Veh = 5; Het, LP-211 = 5. The inset shows representative fEPSPs recorded before TBS and 40 min after TBS. Each trace is the average of three successive fEPSPs (artifacts of stimulation have been truncated). Calibration bars: 0.5 mV, 10 ms.

LTP was induced in CA1 area by TBS delivered to the Schaffer collaterals in hippocampal slices from wt and MeCP2-308 female mice, treated with either LP-211 or vehicle. TBS resulted in a long-lasting increase of fEPSP slope both in wt and in MeCP2-308 female mice (Figure 5). The RM-ANOVA revealed a significant main effect of RM (*F*_(48,912)_ = 8.14; *p* > 0.001) and a significant genotype by treatment interaction (*F*_(1,19)_ = 6.74; *p* = 0.018). *Post hoc* comparisons by Tukey’s test on the genotype by treatment interaction showed a trend towards a reduction in the synaptic potentiation after TBS in vehicle-treated MeCP2-308 females with respect to vehicle-treated wt (Figure 5). A clear effect of the *in vivo* treatment was observed in RTT mice: LP-211 induced a significant increase in synaptic potentiation with respect to vehicle-treated MeCP2-308 females (*p* < 0.05; Figure 5), while the same treatment did not modify the degree of LTP in wt mice.

### Molecular Pathways Related to Protein Synthesis

**Figure 6:**
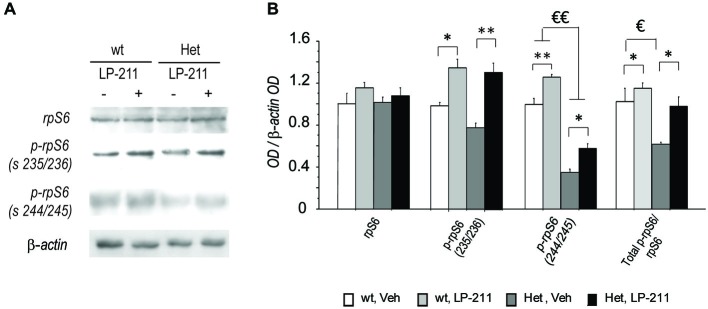
**Long-lasting beneficial effects of the stimulation of the 5-HT7 receptor on RTT-related alterations in rpS6 activity in mouse hippocampus. (A)** Representative Western blot analysis (summarized view corresponding to one animal per group) of rpS6, p-rpS6 Ser 235/236, p-rpS6 Ser 240/244 and β-actin proteins in hippocampi of MeCP2-308 female mice (Het) and wt mice in control conditions (Veh) or treated with LP-211. In order to limit background and unspecific signals, the membranes related to rpS6 and p-rpS6 Ser 240/244 were cut at the opportune kDa range with the help of the MW marker before hybridization. **(B)** Semi-quantitative densitometric analysis, obtained by optical density (OD) of rpS6 signals normalized with OD of β-actin signals. OD ratios are expressed as the average fold increase vs. wt controls. *N* = 4 per each experimental groups. Data are mean ± SEM. €: wt, Veh vs. Het, Veh; *p* < 0.05; *: Veh vs. LP-211, *p* < 0.05; €€: wt, Veh vs. Het, Veh; *p* < 0.01; **: Veh vs. LP-211, *p* < 0.01.

To determine whether the molecular modifications we detected at the end of a seven-day long treatment with LP-211 in the brain of RTT mice persist (De Filippis et al., 2014a), the amount and activation of rp-S6 were analyzed by western blot analyses in the hippocampi collected after a washout period of 2 months from the last (7th) ip injection of either LP-211 or vehicle. The ANOVA did not yield either genotype or treatment effects as for total rpS6 protein level (Figure 6). Phosphorylation levels of the rpS6 was generally reduced in the hippocampus of MeCP2-308 female mice (Figure 6), thus confirming the profile reported in MeCP2-mutated males (Ricciardi et al., 2011; De Filippis et al., 2014a). Such effect was however more marked at Ser240/244, than at Ser235/236 (main effect of genotype: p-rpS6 (235/236): *F*_(1,12)_ = 3.86; *p* = 0.073; p-rpS6 (240/244): *F*_(1,12)_ = 238.03; *p* < 0.001; Figure 6).

Consistently, the ratio between the phosphorylated forms and the total rpS6 protein content, which provides an index of the net functionality of the kinase, was shifted toward decreased phosphorylation (inactivation) in RTT mouse hippocampus compared to wt controls (main effect of genotype: Total p-rpS6/rpS6: *F*_(1,12)_ = 12.75; *p* < 0.001). Of note, LP-211 treatment significantly increased the levels of S6 phosphorylation in both genotypes (main effect of treatment: p-rpS6 (236/238)/rpS6: *F*_(1,12)_ = 46.84; *p* < 0.001; p-rpS6 (240/244)/rpS6: *F*_(1,12)_ = 32.45; *p* < 0.001) and the total p-rpS6/ rpS6 ratio (main effect of treatment: Total p-rpS6/rpS6: *F*_(1,12)_ = 8.99; *p* = 0.011), thus restoring wt-like levels of S6 phosphorylation in RTT mouse brain (Figure 6).

## Discussion

The present study extends our previous findings (De Filippis et al., 2014a) and demonstrates that a seven-day-long stimulation of the 5-HT7 receptor significantly improves RTT-related impairments in spatial reference memory and synaptic plasticity, observed in symptomatic female mice modeling RTT. In addition, a delayed beneficial effect of the LP-211 treatment on general health status was also discovered in MeCP2-308 Het female mice. Notably, treatment evaluation on behavioral and molecular parameters was carried out up to 2 months after the last injection. The present study thus provides evidence of long-lasting beneficial effects of a transient LP-211 exposure on RTT-related impairments.

Consistent with previous studies in males (Moretti et al., 2006; De Filippis et al., 2010), we found that MeCP2-308 female mice show synaptic plasticity deficits, cognitive and motor coordination impairment as well as slight, but reliable phenotypic alterations. The present results in females confirm that neurobehavioral alterations are detectable under conditions of heterozygosis in RTT mouse models (Katz et al., 2012; Samaco et al., 2013). As the estrous cycle was not controlled in this study, we cannot completely exclude the possibility that cycle genotype differences may have exerted a role, although this seems unlikely (see the review paper: Prendergast et al., 2014). Of note, all the alterations we uncovered in RTT female mice were counteracted by a seven-day long treatment with LP-211. These include the phenotypic alterations, the impairments in cognitive and synaptic plasticity, as well as motor coordination deficits. Taken together with our previous study (De Filippis et al., 2012), these results provide evidence that a LP-211 treatment exerts a widespread beneficial effect on RTT-related symptomatology in a mouse model. Moreover, our data demonstrate that the agonist administration is equally effective when tested in the gender and the hormonal milieus which are more relevant for RTT (Katz et al., 2012), certainly increasing the translational value of the present study.

Our results provide further evidence that stimulation of 5-HT7 receptors acts consistently onto spatial memory and synaptic plasticity (Roberts and Hedlund, 2012; Volpicelli et al., 2014). As a whole, these data add to a complex picture in which 5-HT7 receptor agonists and antagonists have been reported to have both promnesic and/or anti-amnesic effects (Meneses et al., 2015), with major results pointing to an involvement of the 5-HT7 receptor in spatial memory (Hedlund, 2009; Sarkisyan and Hedlund, 2009; Beaudet et al., 2015). Interestingly, we found that the effects of the LP-211 treatment strongly depend on the basal level of performance, thus confirming previous studies (Meneses et al., 2015). In the Barnes Maze test, for instance, we found that the LP-211 treatment improved the defective performance of RTT mutants, while dampening the spatial memory of normal mice. This feature is certainly of high relevance in a translational context, in which a normalization of abnormal performances represents the most attractive outcome.

Interestingly, the beneficial effects of the treatment over the general health status of RTT mice became evident 3 weeks after the last administration of LP-211, possibly as a result of stimulated neuroplasticity. In other words, some weeks may be needed before the consequences of 5-HT7R stimulation appear, in the form of a general improvement of RTT-related neurological alterations. In particular, the phenotypic parameters which appeared to be improved in treated RTT mice include the fur, the abnormal gait, the kyphosis and the breathing abnormalities. Further studies are however needed to verify whether such effect results from an improvement in those autonomic functions which are related to aberrant serotonergic signaling in RTT, such as the respiratory brainstem dysfunction (Abdala et al., 2010, 2014). Plethysmograpic analyses will certainly help shedding light on this possibility and to corroborate our general health scoring.

By contrast, a seven-day-long treatment with LP-211 appeared to provoke a transient worsening of the general condition of wt mice and of their performance in the *Dowel test* and the *Barnes Maze task*. Phenotypic features which appeared most compromised in LP-211-treated wt mice include mobility and fur. Interestingly, we have previously reported that sub-chronic treatment with LP-211 reduces the 5-HT7R expression in wt mouse hippocampus, to levels comparable to those found in RTT mouse brain (De Filippis et al., 2014a). Note however that present wt mice were aged 1 year, and that a comparable treatment during adolescent age resulted in a potentiation of forebrain connectivity to the hippocampus, of 5-HT7R function in the septum as well as of spatial memory skills (Altabella et al., 2014; Canese et al., 2015). It will be interesting to determine whether the direction of LP-211 effects may depend on age, and whether modulated expression of the 5-HT7R in rodent brains may account for the phenotypic effects of a subchronic LP-211 treatment.

One major conclusion of our study concerns the enduring sequelae exerted by a seven-day-long treatment with LP-211 in RTT mice. Even though further studies are needed to uncover the neurobiological mechanisms underlying such effects, we argue that they may have been mediated by LP-211-induced activation of Rho GTPases, a family of proteins crucially involved in intellectual disability disorders (Ramakers, 2002; De Filippis et al., 2014b). Our previous data do in fact demonstrate that the effects of a single intra-cerebro-ventricular injection with CNF1, a bacterial protein known to transiently activate Rho GTPases, were still well evident after months in a RTT mouse model (De Filippis et al., 2012). In the same study, we unequivocally demonstrated the crucial role played by Rho GTPases: the abolition of the CNF1 activity over the activation status of Rho GTPases was sufficient to prevent the beneficial effects of this focal CNF1 treatment.

Rho GTPases are a family of proteins which plays a crucial role in the regulation of synaptic plasticity, synaptogenesis and dendritic spine formation (Luo, 2000; Etienne-Manneville and Hall, 2002; Hall, 2005). Hence, it may be speculated that plastic remodeling of the neural circuitries might account, in RTT mouse brains, for the apparent long-lasting effects of pharmacological approaches targeting Rho GTPases, like CNF1 and LP-211 (Diana et al., 2007; Cerri et al., 2011; De Filippis et al., 2012; Loizzo et al., 2013). In this line, we have recently demonstrated that a sub-chronic treatment with LP-211 during adolescence, a time window of increased plasticity of the central nervous system (Adriani et al., 2006), induces a persistent rearrangement of neural circuitries in rats (Altabella et al., 2014). A similar neuroplastic effect might thus account for the long-lasting beneficial effects of LP-211 we presently report in RTT mice. Further studies are however needed to clarify this point.

Notably, we found that the molecular effects of LP-211 treatment over the activation status of the rpS6, the downstream target of mTOR and S6 kinase responsible for the altered protein translational control in RTT mouse brain (Ricciardi et al., 2011), were still well evident after 2 months from the last injection of LP-211 in RTT mouse hippocampus. Even though the relative role of these molecular effects at the phenotypic level is still unclear, such results are particularly interesting as the normalization of this signaling pathway in an *in vitro* RTT human model is known to rescue disease-related cellular impairments (Li et al., 2013). We cannot at the moment explain how this persistent molecular effect is achieved, and further studies are needed. We can, however, certainly exclude that it can be related to brain accumulation of LP-211 or to persistent binding to the 5-HT7R, based on previous studies demonstrating that LP-211 is no longer detectable in mouse brain after 120 min from a single (10 mg/kg, ip) injection (Leopoldo et al., 2008).

## Conclusion

Overall, the present study extends previous findings from our laboratory, also highlighting persistent effects, and provides compelling preclinical evidence of the potential therapeutic value of a pharmacological approach targeting the brain serotonin receptor 7. This innovative approach may turn out to be relevant for RTT, a devastating disorder for which no cure is currently available. The high potential of innovative drugs, capable of targeting the activation status of the Rho GTPases family, is also further supported. Given the promising results so far obtained at the preclinical level, any effort to narrow the gap between preclinical and clinical research is now urgent and mandatory.

## Author Contributions

Design of the work: BDF, MRD, AF, GL; Synthesis of LP-211: EL, ML; Data acquisition and analysis: BDF, VC, MRD, AF; Interpretation of data: All authors; Manuscript preparation: BDF; Revision of the manuscript: All authors.

## Conflict of Interest Statement

The authors declare that the research was conducted in the absence of any commercial or financial relationships that could be construed as a potential conflict of interest.
